# Left-sided portal hypertension caused by idiopathic splenic vein stenosis improved by splenectomy: a case report

**DOI:** 10.1186/s40792-020-00912-y

**Published:** 2020-06-26

**Authors:** Hikaru Hayashi, Akira Shimizu, Hiroaki Motoyama, Koji Kubota, Tsuyoshi Notake, Tomohiko Ikehara, Koya Yasukawa, Akira Kobayashi, Yuji Soejima

**Affiliations:** Division of Gastroenterological, Hepato-Biliary-Pancreatic, Transplantation and Pediatric Surgery, Department of Surgery, Shinshu University School of Medicine, 3-1-1 Asahi, Matsumoto, Nagano, 390-8621 Japan

**Keywords:** Left-sided portal hypertension, Idiopathic splenic vein stenosis, Splenectomy

## Abstract

**Background:**

Splenic vein stenosis and occlusion, which are known causes of left-sided portal hypertension, often occur secondary to trauma, pancreatitis, or invasion or compression by pancreatic tumors. However, few reports have described idiopathic splenic vein stenosis.

**Case presentation:**

A 70-year-old man was referred to our hospital for examination of isolated gastric varices. He had no history of liver disease, pancreatitis, or abdominal trauma. Computed tomography revealed stenosis of almost the entire length of the splenic vein, and development of gastric fundal and short gastric varices. No inflammatory changes or neoplastic lesions of the pancreas were observed in any imaging study. The patient was diagnosed with left-sided portal hypertension caused by idiopathic splenic vein stenosis, and splenectomy was performed. The postoperative course was smooth, and improvement of the gastric varices was shown by upper gastrointestinal endoscopy at 3 months after the operation.

**Conclusions:**

Idiopathic splenic vein stenosis is an extremely rare cause of left-sided portal hypertension. Splenectomy is one of the most effective treatments for left-sided portal hypertension caused by idiopathic splenic vein stenosis.

## Background

Left-sided portal hypertension (LSPH) is characterized by the flow of splenic venous blood into the portal trunk via the collateral circulation due to narrowing or obstruction of the splenic vein. Although splenic vein stenosis or occlusion is often caused by trauma, pancreatitis, or invasion or compression by a pancreatic tumor, few reports have described idiopathic splenic vein stenosis. Herein, we report a case of LSPH caused by idiopathic splenic vein stenosis.

## Case presentation

**Fig. 1:**
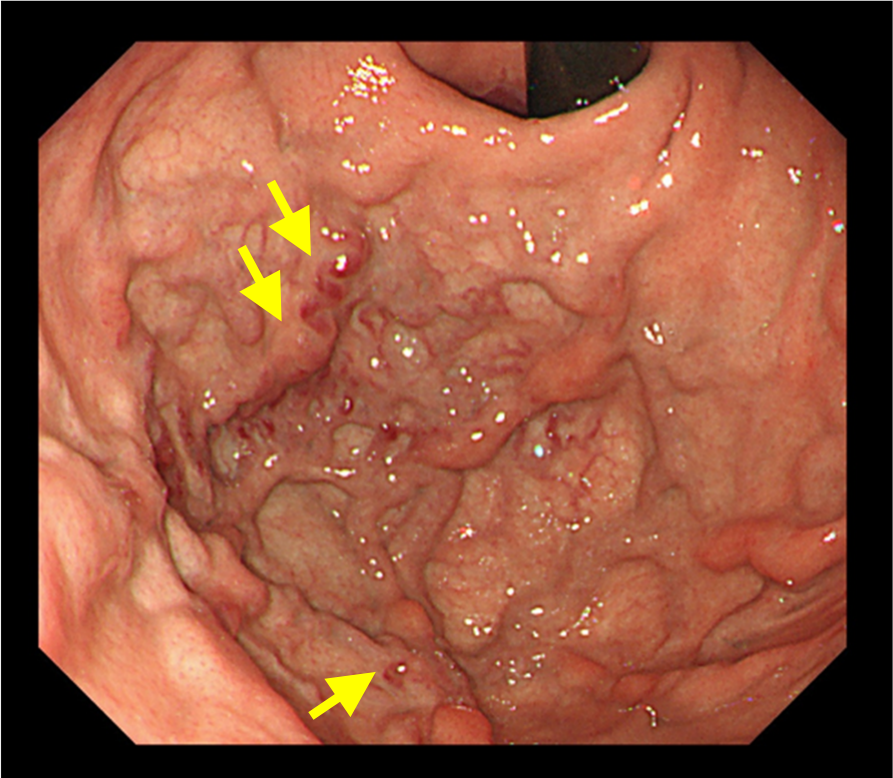
Upper gastrointestinal endoscopy showed isolated varices in the gastric fundus

**Fig. 2:**
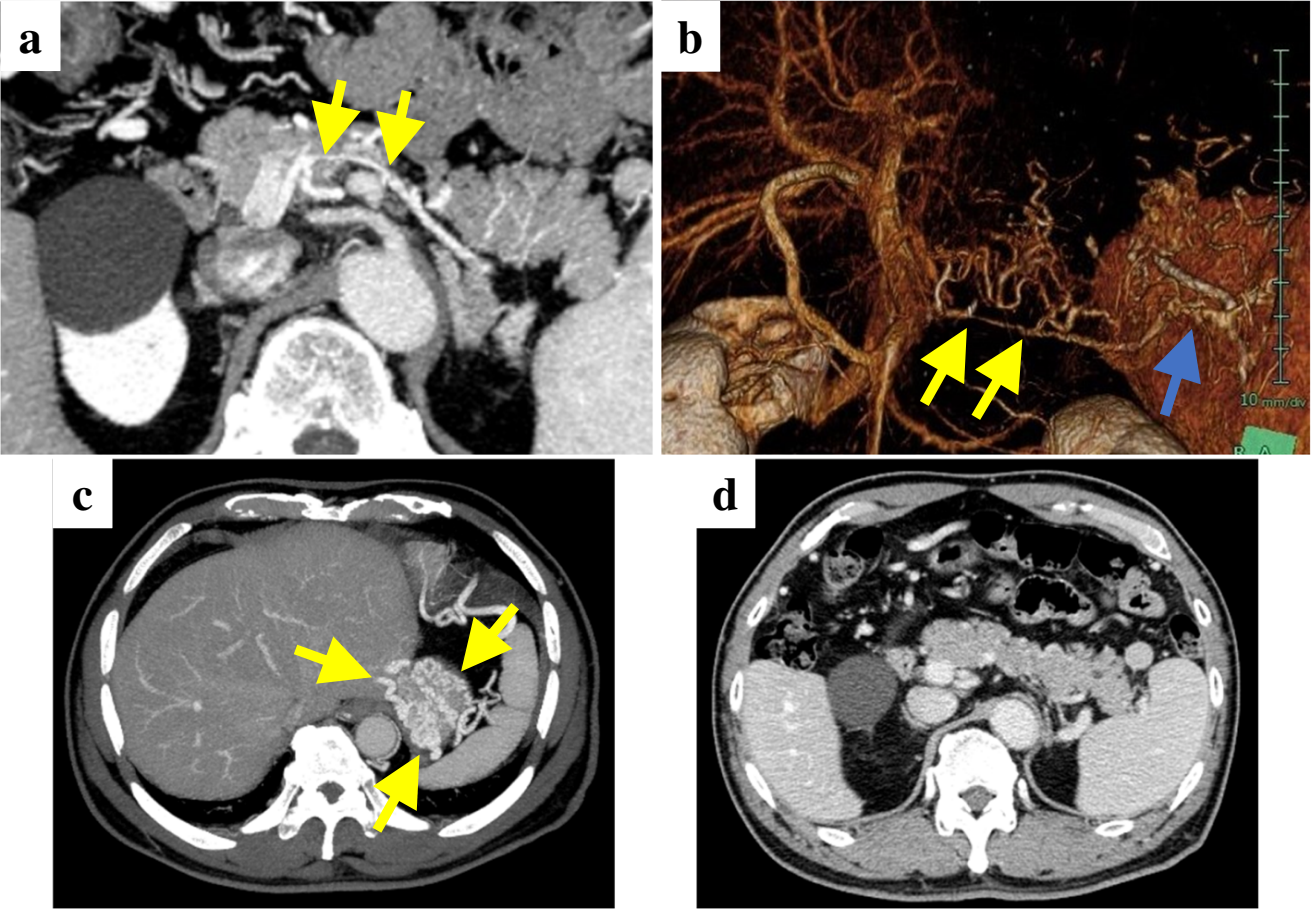
Contrast-enhanced abdominal computed tomography findings. **a**, **b** Stenosis (2 mm in diameter, yellow arrows) of almost the entire length of the splenic vein, with post-stenosis dilatation at the splenic hilum (10 mm in diameter, blue arrows). **c** Development of gastric fundal varices and short gastric veins. **d** No inflammatory changes around the pancreas or pancreatic neoplasm were observed. **a**, **c**, **d** Axial views. **b** Blood vessel construction image

A 70-year-old man presented with the chief complaint of epigastric discomfort. Upper gastrointestinal endoscopy showed isolated gastric varices (Fig. 1), and the patient was referred to our hospital for further examination. He had no history of major organ diseases that can cause splenic vein stenosis, such as pancreatitis or abdominal trauma. The liver was impalpable, and no obvious abdominal tenderness or rebound pain was elicited. The spleen was also impalpable, and no signs of liver cirrhosis such as jaundice or superficial vein engorgement were found. Laboratory data showed a white blood cell count of 6050 cells/μL, normal differential counts, a C-reactive protein concentration of 0.07 mg/dL, a slightly low platelet count of 139,000 cells/μL, normal liver function and coagulation tests, and unremarkable tumor marker concentrations. Abdominal computed tomography (CT) revealed stenosis (2 mm in diameter) of almost the entire length of the splenic vein, with post-stenosis dilatation at the splenic hilum (10 mm in diameter; Fig. 2a, b), and development of gastric fundal varices and short gastric veins (Fig. 2c). No inflammatory changes around the pancreas or pancreatic neoplasm were observed in any imaging study (Fig. 2d). Additionally, the left gastric vein (LGV) was highly contrasted (to the same extent as the portal vein) before the superior mesenteric vein was completely contrasted. Therefore, the patient was considered to have hepatopetal venous blood flow in the LGV.

**Fig. 3:**
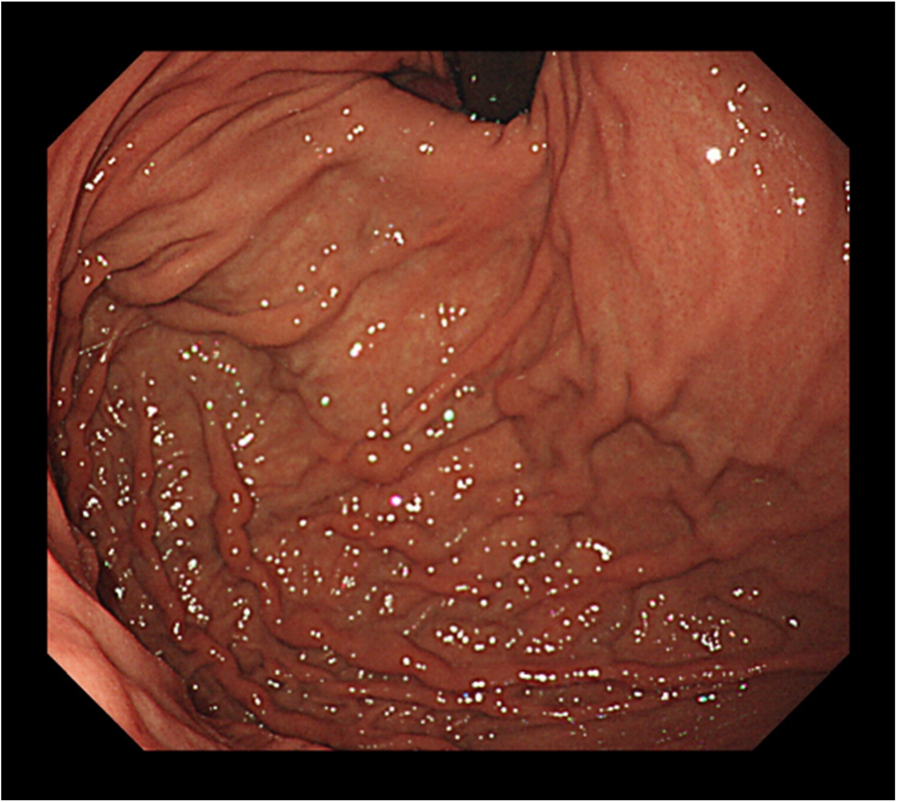
Upper gastrointestinal endoscopy at 3-months postoperatively showed disappearance of the gastric varices

Based on these findings, we diagnosed the patient with LSPH caused by idiopathic splenic vein stenosis. Splenectomy was performed because of concerns about the risk of bleeding from the gastric varices. No obvious abnormality was detected around the splenic vein. The liver surface was smooth and the edges were sharp, while the spleen weighed 120 g and was macroscopically almost normal. Histopathological examination showed slightly conspicuous sinus hyperplasia, but no obvious findings of neoplastic lesions or vasculitis, including around the splenic vein. Congo red staining was also performed, but no obvious amyloid deposits were observed. The postoperative course was smooth, and upper gastrointestinal endoscopy performed 3 months after the operation showed that the gastric varices had disappeared (Fig. 3).

## Discussion

LSPH is caused by increased venous pressure due to splenic vein stenosis or occlusion. LSPH is considered to be different from other types of portal hypertension, in that it is associated with normal liver function and a patent extrahepatic portal vein. Although the incidence of LSPH has increased over the past decades, and extrahepatic portal vein occlusion itself is reported in 340–560 patients per year [1], the percentage of splenic vein stenosis remains unclear. Splenic vein stenosis results in venous hypertension in the collateral pathways that carry splenic arterial blood to the veins located in the upper half of the stomach. In the gastric wall veins of the fundus, blood flow and pressure of the short gastric veins increase and the submucosal structures consequently dilate, which produces gastric varices [2, 3].

The age at definitive LSPH diagnosis is most often < 20 years, followed by the 40s–50s, and the peak age is bimodal. The 10-year cumulative survival rate is 93.3%; that is, splenic vein stenosis has a favorable prognosis. However, treatment for the associated symptoms of portal hypertension is important. The main cause of splenic vein stenosis is splenic vein thrombosis and thrombus formation accompanying the spread of inflammation from acute pancreatitis or chronic pancreatitis. Moossa and Gadd reported [4] that among 144 cases of splenic vein thrombosis, 60% (87 cases) were caused by pancreatitis, and 9% (13 cases) were caused by malignant pancreatic tumors. Other causes of splenic vein stenosis include a pancreatic pseudocyst, migratory spleen, exclusion due to enlargement of nearby lymph nodes, splenic aneurysm, and retroperitoneal disease [5, 6]. Our patient had few notable findings consistent with splenic vein stenosis in his history, preoperative imaging findings, intraoperative findings, or macroscopic and pathological findings of the excised specimens. Thus, we considered that he had idiopathic splenic vein stenosis.

To the best of our knowledge, only two reports to date have described LSPH caused by idiopathic splenic vein stenosis. Suhocki et al. reported [7] the first case of idiopathic splenic vein stenosis. In that study, the initial symptom was hematemesis melena, while CT revealed no adjacent mass formation around the splenic vein, and liver biopsy showed no evidence of hepatitis or cirrhosis. The diagnosis of LSPH caused by idiopathic splenic vein stenosis was made by transhepatic portal and splenic venography with pressure measurements, which revealed a focal stenosis of the splenic vein near the splenic hilum, with a post-stenotic dilatation and short gastric veins draining into the LGV through the gastric wall varices. Splenectomy was performed as a curative measure, and there was no recurrence during the following period. Addario et al. also reported [8] a case of LSPH during pregnancy caused by segmentary idiopathic splenic vein stenosis. In that study, the patient presented to the emergency department with hematemesis melena and hypovolemic shock, while CT showed an abnormal splenic vein with a sharp lumen structure and post-stenotic dilation, with a normal liver and an enlarged spleen. Because no cause of acquired splenic vein stenosis could be identified, the authors hypothesized that the origin was congenital. Hemostasis was achieved by endoscopic injection of acrylate glue into the bleeding varices; the patient remained stable after the procedure, and an urgent cesarean section was performed with successful delivery of the baby. Because of the high risk of recurrent bleeding, the patient was advised to undergo either percutaneous or surgical angioplasty and stenting, and/or splenectomy. However, she refused any intervention, and although most of the gastric varices remained, she was in good health during the following 2 years.

To summarize these two reports, the definitions of LSPH caused by idiopathic splenic vein stenosis were as follows: (1) stenosis of the splenic vein with post-stenotic dilatation; (2) no evidence of a cause of splenic vein stenosis, such as pancreatitis, pancreatic tumor, cysts, thrombosis, arterial aneurysm, retroperitoneal fibrosis, or iatrogenic injury; (3) normal liver function tests, normal appearance of the liver by imaging studies, and/or no evidence of hepatitis or cirrhosis by histological examination; and (4) presence of gastric varices and multiple collateral vessels leading from the spleen to the stomach via the short gastric veins with hepatopetal blood flow. In the present case, although the first event was different, the diagnostic method was the same. Regarding treatment, although splenectomy was performed in the first case, while endoscopic treatment was performed in the pregnant case, surgical treatment would have been performed if this patient had consented.

Gastric varices are generally asymptomatic and are often identified incidentally. The most common clinical symptom in symptomatic patients with LSPH is gastrointestinal bleeding from a ruptured gastric varix. Takagi et al. reported [9] that the rate of bleeding from gastric varices caused by splenic vein stenosis was approximately 33%. In a study of LSPH [2], 45–72% of patients reported the above symptoms, while epigastric discomfort was also a common symptom, as in the present case. According to the diagnosis and treatment guidelines for aberrant portal hemodynamics [1], endoscopic hemostasis should be performed for cases of gastric variceal bleeding, and endoscopic treatment is performed prophylactically. Yoshida et al. reported [10] that 85% of varicose veins treated with endoscopic varicose nodule surgery or sclerotherapy for gastric variceal bleeding showed improvement and that hemostasis was obtained in 100% of patients. However, it is difficult to reduce the venous pressure of the entire gastric fundus, and there is a contrasting report that the varices could not be controlled using only those procedures [11]. Thus, patients with intractable bleeding and recurrence should undergo surgical treatments such as splenectomy and Hassab’s operation. In a large-scale study of patients with isolated splenic vein thrombosis who underwent splenectomy, no patients developed recurrent gastric varices during the average 11-month postoperative follow-up period [4].

Splenectomy is considered an effective treatment for patients with uncontrollable bleeding. In symptomatic patients, splenectomy is associated with correction of potentially fatal bleeding, prevention of hypersplenism, and a possible reduction or elimination of future bleeding episodes. Wang et al. reported [12] that surgical interventions such as splenectomy may provide a good long-term outcome in symptomatic patients. However, any consensus on the treatment strategy in asymptomatic patients has not been established. Initial observation and conservative management may be acceptable, although the definitive management of this condition traditionally involves surgical removal of the primary cause if possible. Additionally, portal hypertension is associated with secondary hypersplenism, and leukopenia and thrombocytopenia can often occur. Therefore, splenectomy may benefit patients with those conditions.

In any case, the management of LSPH should be directed at the splenic side of the portal circulation, because pressure is increased only on that side [13]. Unlike portal hypertension caused by cirrhosis, proximal portal decompressive procedures are hazardous and do not address the disease process. As endoscopic treatment only provides symptomatic management, the definitive management of this condition involves surgical removal of the primary cause if possible. Splenectomy is considered to decrease the arterial inflow into the left portal system, which results in decompression of the gastric varices [2].

It is also important to consider postoperative complications after splenectomy. Peculiar complications include pancreatic fistula and overwhelming post-splenectomy infection (OPSI) [14, 15], which can be fatal. Pancreatic fistula is not a rare complication, with a prevalence of 4.5–16% in patients with hematological diseases [16, 17]. Furthermore, various infections are more likely to occur after splenectomy because of the diminished immunological response. OPSI is one of the most serious infections caused by an encapsulated pathogen. OPSI occurs in approximately 5% of patients, and the time of onset varies from several days to several years, with a mortality rate of approximately 50%. Therefore, patients should be given sufficient information to be able to provide informed consent, and a pneumococcal vaccine should be given preoperatively. Indeed, Wang et al. reported [18] a reduced incidence of infections in patients with higher rates of adherence to current post-splenectomy guidelines.

In patients for whom surgical treatment is high risk, splenic artery embolization has been reported as an alternative treatment option, and in patients with splenic vein stenosis without occlusion, stent implantation in the splenic vein has been reported. However, these treatments may lead to splenic abscesses or delayed splenic perforation [19], and they should be implemented with caution. In our patient, upper gastrointestinal endoscopy was positive for the red color sign; we were concerned about the high risk of bleeding and required a treatment to prevent fatal bleeding and relieve epigastric discomfort, while endoscopic treatments such as sclerotherapy or clipping were difficult because of the morphology of the varices and the amount of blood flow. The splenic vein was also narrowed over almost its entire length, which made it difficult to implant a stent. Furthermore, thrombocytopenia was observed, while the patient had no comorbidities. Therefore, splenectomy was considered the best treatment.

## Conclusions

We report a case of LSPH caused by idiopathic splenic vein stenosis that was improved by splenectomy. There are few reports of idiopathic splenic vein stenosis, and this condition can sometimes be fatal. Splenectomy is one of the most effective treatments for patients with intractable bleeding and recurrence from gastric varices.

## Data Availability

The dataset supporting the conclusions of this article is included within the article.

## Abbreviations

LSPH: Left-sided portal hypertension
CT: Computed tomography
LGV: Left gastric vein
OPSI: Overwhelming post-splenectomy infection
